# Supplementary material for: WordCommentsAnalyzer: A windows software tool for qualitative research
Source: F1000Res. 2018 Sep 4;7:536. Originally published 2018 May 3. [Version 2] doi: 10.12688/f1000research.14819.2 (PMC6137411; doi:10.12688/f1000research.14819.2)
Supplement: Supplementary file 3 [file f1000research-7-17614-s0002.tgz › b2a79496-4ada-47c4-9682-a99ce867836d.zip!/Eight Iranian Health Journals/Archives of Rehabilitation_2.docx]

© Archives of Rehabilitation (RJ). University of Social Welfare and Rehabilitation Sciences, Koodakyar Alley, Daneshjoo Blv., Evin, Tehran, Iran. This work is licensed under [CC BY-NC 4.0](https://creativecommons.org/licenses/by-nc/4.0/).

Volume & Issue: دوره 18 - شماره 4

Title: بررسی تأثیر پوسچرهای مختلف کاری بر عملکرد شناختی دانشجویان

http://rehabilitationj.uswr.ac.ir/article-1-2013-fa.html

هدف برای انجام فعالیت در محیط کار پوسچرهای مختلفی وجود دارد که می‌تواند بر عملکرد افراد تأثیر بگذارد. در محیط کار اداری، اغلب از پوسچرهای نشسته استفاده می‌شود. پوسچرهای نشسته نامطلوب و ناخواسته، علاوه برایجاد خستگی عضلانی و احساس ناراحتی در ستون فقرات، سبب اعمال بار ذهنی می‌شود. پوسچر نامطلوب و ناخواسته سبب بروز خطای انسانی و تأخیر در فرایند پردازش اطلاعات می‌شود. در تحقیقات اخیر ارتباط میان پوسچر استاتیک و عملکرد شناختی مانند زمان واکنش به محرک‌های شنیداری نشان داده شده است. در میان پژوهش‌ها، مطالعه‌ای که تأثیر هم‌زمان پوسچر ایستاده و نشسته را بر عملکرد شناختی بررسی کرده باشد، وجود ندارد. هدف از این مطالعه، ارزیابی عملکرد شناختی هنگام اجرای وظایف در سه پوسچر مختلف کاری استاتیک ایستاده استاندارد، نشسته استاندارد و نشسته انتخابی بوده است.  روش بررسی مطالعه حاضر به صورت نیمه‌تجربی، روی ۲۹ نفر از دانشجویان سالم با طیف سنی ۲۰ تا ۳۰ سال صورت گرفت. نمونه‌گیری به صورت غیراحتمالی انجام شد. همه آزمودنی‌ها فرم موافقت آگاهانه را تکمیل کردند. نداشتن اختلالات اسکلتی عضلانی، نداشتن بیماری‌های قلبی، نداشتن سابقه جراحی در بخش‌های مختلف ستون فقرات و نداشتن اختلالات افسردگی و اضطراب ازجمله معیارهای ورود به مطالعه بود. به منظور تعیین میزان افسردگی و اضطراب از پرسش‌نامه سنجش افسردگی و اضطراب بک استفاده شد. کمیته اخلاقی دانشگاه علوم بهزیستی و توانبخشی این مطالعه را تأیید کرد. شرکت‌کنندگان در دانشگاه علوم بهزیستی و توانبخشی در شرایط آزمایشگاهی و روی ایستگاه کاری اداری به اجرای آزمون استروپ پیچیده (۹ تا ۱۲ دقیقه تایپ به مدت ۵ دقیقه) پرداختند. پوسچرهای بررسی‌شده ازجمله معمول‌ترین پوسچرهای به‌کارگرفته‌شده در محیط اداری بودند. به منظور بررسی تأثیر پوسچرهای ایستاده استاندارد، نشسته استاندارد و نشسته انتخابی بر متغیرهای وابسته مانند کل زمان آزمون، زمان واکنش به محرک‌های همخوان و ناهمخوان، تعداد کلمات تایپ‌شده و تعداد اشتباهات تایپی، از آزمون تحلیل واریانس با اندازه‌گیری‌های مکرر استفاده شد. همچنین برای بررسی تفاوت موجود در هر یک از گروه‌ها، از آزمون تی زوجی استفاده شد. یافته‌ها نتایج نشان داد متغیر زمان واکنش و دقت آزمودنی‌ها تحت تأثیر پوسچرهای کاری است (۰/۰۰۱=P). نتایج تجزیه‌وتحلیل تعقیبی گویای اختلاف معنی‌دار در مدت‌زمان واکنش میان دو وضعیت بدنی نشسته انتخابی و نشسته استاندارد (۰/۰۰۱=P)، نشسته انتخابی و ایستاده استاندارد (۰/۰۴۳=P) و نشسته استاندارد و ایستاده استاندارد (۰/۰۰۱=P) است. با درنظرگرفتن میانگین مدت‌زمان واکنش برای محرک‌های همخوان و غیرهمخوان، مشخص شد که زمان واکنش در پوسچر نشسته استاندارد (M=۶۲۸/۶۷ms) در مقایسه با پوسچر نشسته انتخابی (M=۶۸۹/۴۱ms) و پوسچر ایستاده استاندارد (M=۶۷۵/۱۶ms) کمتر است. همچنین بین سه پوسچر مطالعه‌شده از نظر تعداد اشتباهات تایپی اختلاف معنی‌داری وجود داشت (۰/۰۰۱=P) . میانگین تعداد اشتباهات تایپی و خطاهای نگارشی در شرایط وضعیت بدنی نشسته استاندارد نسبت به دیگر وضعیت‌ها کمتر بود ( ۰/۵۸=P).  نتیجه‌گیری نتایج این مطالعه نشان داد عملکرد شناختی تحت تأثیر پوسچر قرار می‌گیرد. در میان پوسچرهای کاری بررسی‌شده در این مطالعه، پوسچر نشسته استاندارد از همه بهتر بود. پوسچر نشسته استاندارد می‌تواند به بهبود عملکرد شناختی در محیط کار منجر شود.

Title: مقایسه توجه انتخابی و نیمرخ هوشی در نوجوانان دوزبانه‌ و تک‌زبانه‌

http://rehabilitationj.uswr.ac.ir/article-1-1910-fa.html

هدف با توجه به ارتباط نزدیک میان شناخت و زبان، دوزبانگی می‌تواند توانایی‌های شناختی را تحت تأثیر قرار دهد. هدف پژوهش حاضر مقایسه توجه انتخابی و نیمرخ هوشی در نوجوانان زبان‌آموز مسلط به زبان انگلیسی و نوجوانان غیر زبان‌آموز بود. روش بررسی پژوهش حاضر از نوع علّی مقایسه‌ای است. این پژوهش به روش مقطعی انجام‌ شد. جامعه آماری شامل تمام نوجوانان زبان‌آموز (۱۳ تا ۱۵ ساله) مسلط به زبان انگلیسی و نوجوانان غیر زبان‌آموز شهر اصفهان در سال ۱۳۹۴ بود. به‌ منظور انجام پژوهش، ۶۴ نوجوان در محدوده سنی ۱۳ تا ۱۵ سال با سطح پیشرفته زبان انگلیسی (سطح RECE و REACH در کانون زبان ایرانیان و سطوح معادل آن در آموزشگاه‌های دیگر) به ‌عنوان گروه مورد (زبان‌آموز) و ۵۱ نوجوان غیر زبان‌آموز (تک‌زبانه) در همان رده سنی (۱۳ تا ۱۵ سال) به عنوان گروه شاهد از منطقه ۳ شهر اصفهان انتخاب شدند. نوجوانان هر دو گروه از آموزشگاه‌های مشابه انتخاب شدند. انتخاب نمونه به روش نمونه‌گیری غیرتصادفی در دسترس انجام شد. به منظور بررسی و اندازه‌گیری نیمرخ هوشی نوجوانان زبان‌آموز مسلط به زبان انگلیسی و نوجوانان غیر زبان‌آموز، از پرسش‌نامه هوش چندگانه گاردنر استفاده شد. برای سنجش و اندازه‌گیری توانایی توجه انتخابی در دو گروه نوجوانان زبان‌آموز مسلط به زبان انگلیسی و نوجوانان غیر زبان‌آموز، از نسخه فارسی کامپیوتری‌شده آزمون استروپ کلاسیک استفاده شد. داده‌های به‌دست‌آمده با پرسش‌نامه هوش چندگانه گاردنر و نسخه فارسی کامپیوتری‌شده آزمون استروپ کلاسیک، از طریق روش‌های‌ آماری توصیفی و با استفاده از روش آماری تحلیل واریانس چند متغیری تجزیه‌وتحلیل شد. داده‌ها با استفاده از نسخه ۲۲ نرم‌افزار SPSS تجزیه‌وتحلیل شد. یافته‌ها نتایج نشان داد بین نمره نوجوانان گروه غیر زبان‌آموز و گروه زبان‌آموز در شش مؤلفه هوش چندگانه که عبارتند از: هوش میان‌فردی، موسیقیایی، طبیعت‌گرا (در سطح P<۰/۰۵)، منطقی ریاضی، درون‌فردی و زبانی (در سطح P<۰/۰۰۱) تفاوت معناداری وجود دارد. نتایج پژوهش حاکی از آن بود که نمره نوجوانان زبان‌آموز در این شش مؤلفه هوش چندگانه به‌ طور معناداری بالاتر از همتایان غیر زبان‌آموز بود؛ به‌عبارت‌دیگر، نوجوانان زبان‌آموز در این شش مؤلفه در مقایسه با همتایان غیر زبان‌آموز خود نیمرخ هوشی بهتری داشتند. همچنین بین عملکرد دو گروه در متغیر توجه انتخابی (مرحله سوم آزمون استروپ کلاسیک) تفاوت معناداری وجود داشت (P<۰/۰۵). زمان واکنش نوجوانان زبان‌آموز در تکالیف استروپ کلاسیک کمتر از نوجوانان غیر زبان‌آموز بود. این نتیجه حاکی از سرعت‌عمل آن‌ها در این آزمون و درنتیجه عملکرد بهتر نوجوانان زبان‌آموز نسبت به همتایان غیر زبان‌آموز در آزمون استروپ کلاسیک است. به‌طورکلی نتایج مطالعه حاضر را می‌توان چنین خلاصه کرد که نوجوانان زبان‌آموز در تکالیف مربوط به توجه انتخابی، مؤلفه‌های هوش منطقی ریاضی، زبانی، میان‌فردی، موسیقیایی، درون‌فردی و طبیعت‌گرا بهتر از همتایان غیر زبان‌آموز خود عمل کردند. نتیجه‌گیری بر اساس یافته‌های پژوهش می‌توان نتیجه گرفت که فراگیری زبان دوم (انگلیسی) ممکن است بر توجه انتخابی و نیمرخ هوشی نوجوانان تأثیرگذار باشد. ازاین‌رو نقش آن در توجه انتخابی و هوش نوجوانان باید مدنظر قرار گیرد.

Title: بررسی ارتباط ارزشیابی عملکرد با انگیزش شغلی درمانگران مراکز توانبخشی و بیمارستان‌های دولتی تهران بر مبنای الگوی دوعاملی هرز برگ

http://rehabilitationj.uswr.ac.ir/article-1-1871-fa.html

هدف در حیات هر سازمان عوامل مختلفی نقش دارد که در بین این عوامل مهم‌ترین عامل، نیروی انسانی است. نیروی انسانی باانگیزه برای انجام وظایف، اساسی‌ترین عامل بهره‌وری است. سیستم ارزشیابی عملکرد کارکنان، به‌عنوان ابزاری برای رشد و ارتقای کیفیت وکمیّت عملکرد منابع انسانی به‌شمار می‌آید. ارزشیابی عملکرد، بخشی از روند نظارتی مدیریت محسوب می‌شود. این مطالعه با هدف بررسی ارتباط ارزشیابی عملکرد با انگیزش شغلی درمانگران توان‌بخشی برمبنای الگوی دو عاملی هرزبرگ انجام شد. روش بررسی پژوهش حاضر مطالعه‌ای توصیفی‌تحلیلی بود که به‌صورت مقطعی در سال ۱۳۹۴ روی ۸۰ نفر از درمانگران توان‌بخشی شاغل در مراکز توان‌بخشی و بیمارستان‌های دولتی تهران انجام گرفت. روش نمونه‌گیری پژوهش حاضر، نمونه‌گیری خوشه‌ای بود و ابزار گردآوری این پژوهش را دو پرسش‌نامه استاندارد ارزشیابی عملکرد کارکنان و انگیزش شغلی ۴۰ ماده‌ای هرزبرگ تشکیل می‌داد. قبل از هرگونه تحلیل، طبیعی‌بودن متغیرهای مدنظر با آزمون کولموگروف‌اسمیرنف بررسی شد. سپس با مشاهده طبیعی‌بودن متغیرها، داده‌ها با استفاده از آمار توصیفی، آزمون تی مستقل و ضریب همبستگی پیرسون در نرم‌افزار SPSS تجزیه‌وتحلیل شد. یافته‌ها یافته‌های پژوهش حاضر نشان داد که ۹۰ درصد از درمانگران توان‌بخشی کل جامعه پژوهش حاضر ارزشیابی عملکرد سازمان را ضروری دانستند و ۸/۸۸ درصد از درمانگران اظهار داشتند که ارزشیابی عملکرد بر انگیزش شغلی آن‌ها مؤثر است. این در حالی است که ۵/۵۷ درصد از درمانگران پژوهش، روند فعلی نمره‌دهی ارزشیابی عملکرد را نمی‌پسندیدند. در پژوهش حاضر، ارتباط ارزشیابی عملکرد با انگیزش شغلی درمانگران معنادار نشد (P=۰/۴۷). حیطه عوامل انگیزشی هرزبرگ دربرابر عوامل بهداشتی اهمیت بیشتری داشت؛ براین‌اساس مطالعه حاضر در راستای نتایج تحقیقات هرزبرگ بود. از نظر درمانگران توان‌بخشی پژوهش، در بین عوامل انگیزشی (درونی) هرزبرگ به‌ترتیب عوامل ماهیت کار و مسئولیت شغلی و موقعیت شغلی و در بین عوامل بهداشتی (بیرونی) هرزبرگ، عوامل امنیت شغلی و حقوق و دستمزد بیشترین نقش را در ایجاد انگیزش و عملکرد مؤثر داشت. براساس نتایج حاصل از آزمون‌های آماری، در مطالعه حاضر بین متغیرهای جمعیت‌شناختی از قبیل جنسیت، سطح تحصیلات، وضعیت تأهل، محل خدمت و رشته تحصیلی درمانگران توان‌بخشی با ارزشیابی عملکرد، ارتباط معنادار وجود نداشت. بااین‌حال بین متغیر سابقه کار درمانگران با ارزشیابی عملکرد و عوامل انگیزشی نظریه هرزبرگ رابطه معنادار به‌دست آمد (P=۰/۰۴). همچنین براساس نتایج، ارتباط بین نوع استخدام درمانگران توان‌بخشی و ارزشیابی عملکرد معنادار بود (P<۰/۰۰۶). همچنین بین سن و انگیزش شغلی نظریه هرزبرگ در کل درمانگران جامعه ارتباط معنادار وجود داشت (P=۰/۰۲). نتیجه‌گیری به‌نظر می‌رسد با درنظرگرفتن سختیِ کار درمانگران توان‌بخشی، برای ارتقای سطح انگیزش آن‌ها باید به عوامل انگیزشی مبتنی‌بر نظریه هرزبرگ توجه بیشتری شود. علاوه‌براین ضروری است برای دستیابی به اهداف سازمانی با بهره‌گیری از استانداردها و معیارهای ارزشیابی عملکرد، آموزش‌های لازم به سرپرستان و مسئولان و مدیران داده شود تا آنان عملکرد را به‌طورمطلوب و اثربخش ارزیابی کنند. این امر زمینه‌های افزایش انگیزش شغلی درمانگران توان‌بخشی و پیشرفت کاری و ایجاد دلگرمی بیشتر آنان را فراهم می‌سازد.
